# Oral contraceptives and primary liver cancer.

**DOI:** 10.1038/bjc.1989.94

**Published:** 1989-03

**Authors:** C. La Vecchia, E. Negri, F. Parazzini

**Affiliations:** Mario Negri Institute for Pharmacological Research, Milan, Italy.


*Br. J. Cancer* (1989), **59**, 460–461 © The Macmillan Press Ltd., 1989

### SHORT COMMUNICATION

### Oral contraceptives and primary liver cancer

C. La Vecchia[1,2], E. Negri[1] & F. Parazzini[1]

[1]*Mario Negri Institute for Pharmacological Research, Via Eritrea, 62-20157 Milan, Italy and* [2]*Institute of Social and Preventive Medicine, University of Lausanne, 1005 Lausanne, Switzerland.*

Several anedoctal reports (Sricker & Spoelestra, 1985), analyses of vital statistics (Forman *et al.*, 1983) and at least three case-control studies conducted in California (Henderson *et al.*, 1983) and Britain (Forman *et al.*, 1986; Neuberger *et al.*, 1986) have suggested that the risk of hepatocellular carcinoma is increased in long-term users of oral contraceptives (OC). This association is biologically plausible, since the pill is known to increase substantially the risk of adenomas of the liver (Mettlin & Natarajan, 1981), and experimental evidence has shown that oral contraceptives are effective promoters of hepatocarcinogenesis in rodents (Yager & Yager, 1980).

In Italy, the prevalence of OC use has been traditionally low (La Vecchia *et al.*, 1986). However, inspection of death certification data indicated that the rise in mortality from liver cancer between the late 1950s and the late 1970s was greater in females than in males below age 35, but the opposite was true at later ages (Decarli & La Vecchia, 1984). Although only about 10 deaths per year in Italian women are attributed to primary liver cancer before age 35, and fewer than 50 below age 45, this pattern of trends prompted us to examine further the relation between oral contraceptives and primary liver cancer, using data from a case-control study conducted in Milan.

The data were derived from an ongoing case-control study of several digestive-tract neoplasms, based on a network of teaching and general hospitals in the greater Milan area (La Vecchia *et al.*, 1987). Between January 1984 and October 1987, 21 female cases of histologically or serologically confirmed primary liver cancer (aged 32–59, median age 50) were interviewed using a structured questionnaire including information on socio-demographic indicators, personal characteristics and habits, selected dietary information, a problem-oriented medical history, and history of use of oral contraceptives and other selected drugs.

The comparison group consisted of 145 women (aged 30–59, median age 50), admitted to a network of hospitals with a catchment area comparable to that of cancer cases, for a wide spectrum of acute, non-neoplastic diseases (37% traumas, 13% other orthopaedics, 40% surgical, 10% other miscellaneous).

Statistical analyses were based on standard methods for case-control studies (Breslow & Day, 1980).

The major findings in relation to oral contraceptive use are given in Table I. There were four (19.0%) cases who had ever used OCs compared with 11 (7.6%) controls. The relative risk was 1.8 for up to 5 years' use, and 8.3 for over 5 years. The trend in risk was statistically significant.

**Table I** Relative risk of primary liver cancer in relation to use of oral contraceptives, Milan, Italy, 1984–7

| *Duration of oral contraceptive use (years)* | *Hepatocellular carcinoma* | *Controls* | *Relative risk estimates (95% CI)* |
|---|---|---|---|
| Never used | 17 | 134 | 1 |
| ⩽5 years | 2 | 9 | 1.8 (0.4 and 9.2) |
| >5 years | 2 | 2 | 8.3 (1.4 and 48.7) |
| $\chi_1^2$ (trend) | | | 4.88 ($P$=0.03) |

[a]Adjusted for age in decades.

Hepatitis B serum markers were not determined in this study. A clinical history of hepatitis infection was reported by three (14%) cases and 10 (7%) controls. One case and two controls had evidence of liver cirrhosis. These and other major risk factors for liver cancer in both sexes were considered in a separate paper (La Vecchia *et al.*, 1988).

The epidemiology of primary liver cancer in Italy differs from that in Northern European and American countries, Italy having a higher incidence and mortality, probably in consequence of greater prevalence of its major risk factors, i.e. hepatitis B virus and alcohol consumption (La Vecchia & Decarli, 1985; La Vecchia *et al.*, 1988). It is thus interesting to find confirmation in this population of the association between oral contraceptives and hepatocellular carcinoma, although the small absolute numbers and the low prevalence of ever users were major limitations of this study, impeding any analysis of subgroups or interactions. The low prevalence of pill users even among the cases, furthermore, indicates that, although the relative risk was significantly elevated in long-term users, the attributable risk for oral contraceptives is probably small in this population.

This work was conducted within the framework of the CNR (Italian National Research Council) Applied Project 'Oncology' (Contract No. 87.01544.44). The contribution of the Italian League Against Tumours and of the Italian Association for Cancer Research, Milan, Italy are gratefully acknowledged. We wish to thank Ms Judy Baggott, Gigliola Brambilla Pisoni and the G.A. Pfeiffer Memorial Library for editorial assistance.

### References

BRESLOW, N.E. & DAY, N.E. (1980). *Statistical Methods in Cancer Research, Volume 1, The Analysis of Case-control Studies.* IARC Sci. Publ. IARC: Lyon.

DECARLI, A. & LA VECCHIA, C. (1984). Cancer mortality in Italy, 1955–78. La mortalità per tumori in Italia, 1955–78. *Tumori,* **70,** suppl., 579.

FORMAN, D., DOLL, R. & PETO, R. (1983). Trends in mortality from carcinoma of the liver and the use of oral contraceptives. *Br. J. Cancer,* **48,** 349.

FORMAN, D., VINCENT, T.J. & DOLL, R. (1986). Cancer of the liver and the use of oral contraceptives. *Br. Med. J.,* **292,** 1355.

HENDERSON, B.E., PRESTON-MARTIN, S., EDMONDSON, H.A., PETERS, R.L. & PIKE, M.C. (1983). Hepatocellular carcinoma and oral contraceptives. *Br. J. Cancer,* **48,** 437.

LA VECCHIA, C. & DECARLI, A. (1985). Trends in cancer mortality in Italy, 1955–1978. *Tumori,* **71,** 201.

Correspondence: C. La Vecchia.
Received 22 June 1988, and in revised form, 5 October 1988.

LA VECCHIA, C. & DECARLI, A., PARAZZINI, F., GENTILE, A., NEGRI, E. & FRANCESCHI, S. (1986). Determinants of oral contraceptive use in Northern Italy. *Contraception,* **34,** 145.

LA VECCHIA, C., NEGRI, E., DECARLI, A., D'AVANZO, B. & FRANCESCHI, S. (1987). A case-control study of diet and gastric cancer in Northern Italy. *Int. J. Cancer,* **40,** 484.

LA VECCHIA, C., NEGRI, E., DECARLI, A., D'AVANZO, B. & FRANCESCHI, S. (1988). Risk factors for hepatocellular carcinoma in Northern Italy. *Int. J. Cancer,* **42,** 872.

METTLIN, C. & NATARAJAN, N. (1981). Studies on the role of oral contraceptive use in the etiology of benign and malignant liver tumors. *J. Surg. Oncol.,* **18,** 73.

NEUBERGER, J., FORMAN, D., DOLL, R. & WILLIAMS, R. (1986). Oral contraceptives and hepatocellular carcinoma. *Br. Med. J.,* **292,** 1355.

SRICKER, B.H. & SPOELESTRA, P. (1985). Drug induced hepatic injury. In *Drug Induced Disorders, Vol 1,* Dukes, M.N.G. (ed) p. 237. Elsevier: Amsterdam.

YAGER, J.D. JR. & YAGER, R. (1980). Oral contraceptive steroids as promoters of hepatocarcinogenesis in female Sprague-Dawley rats. *Cancer Res.,* **40,** 3680.


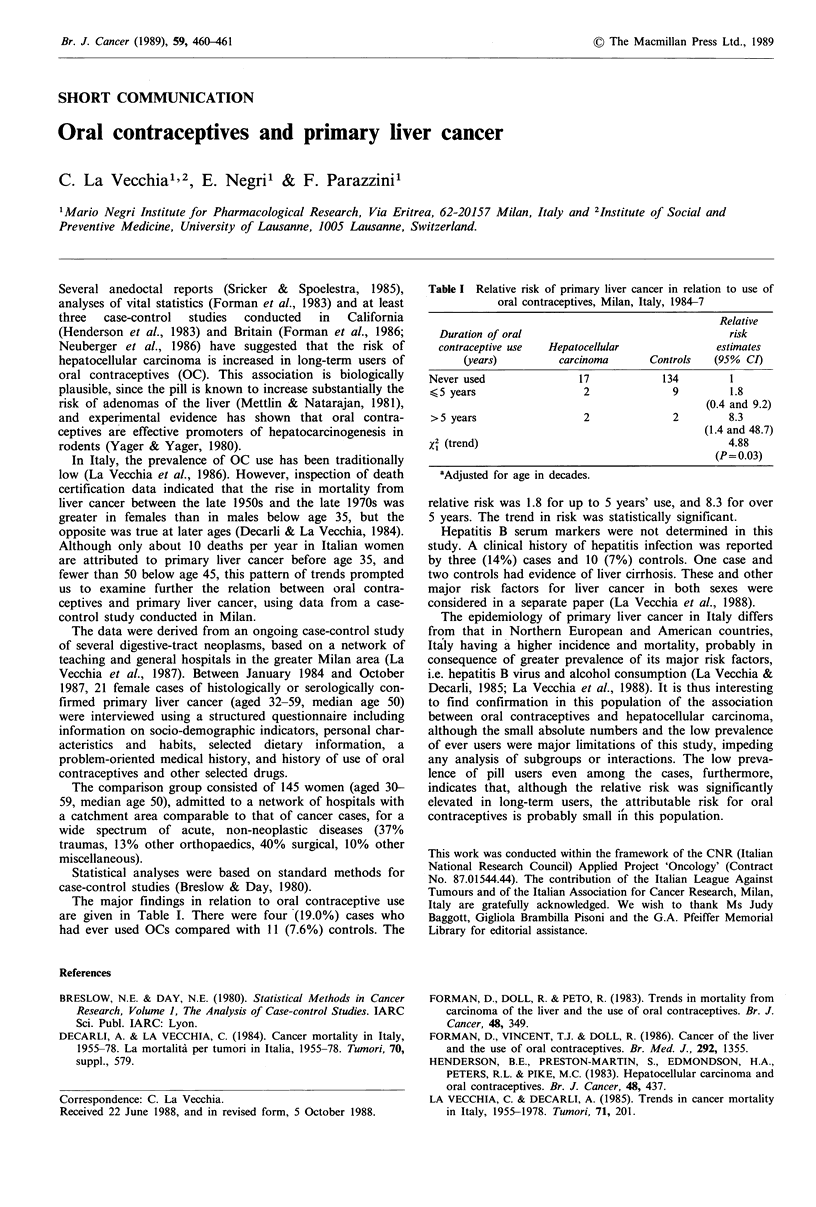

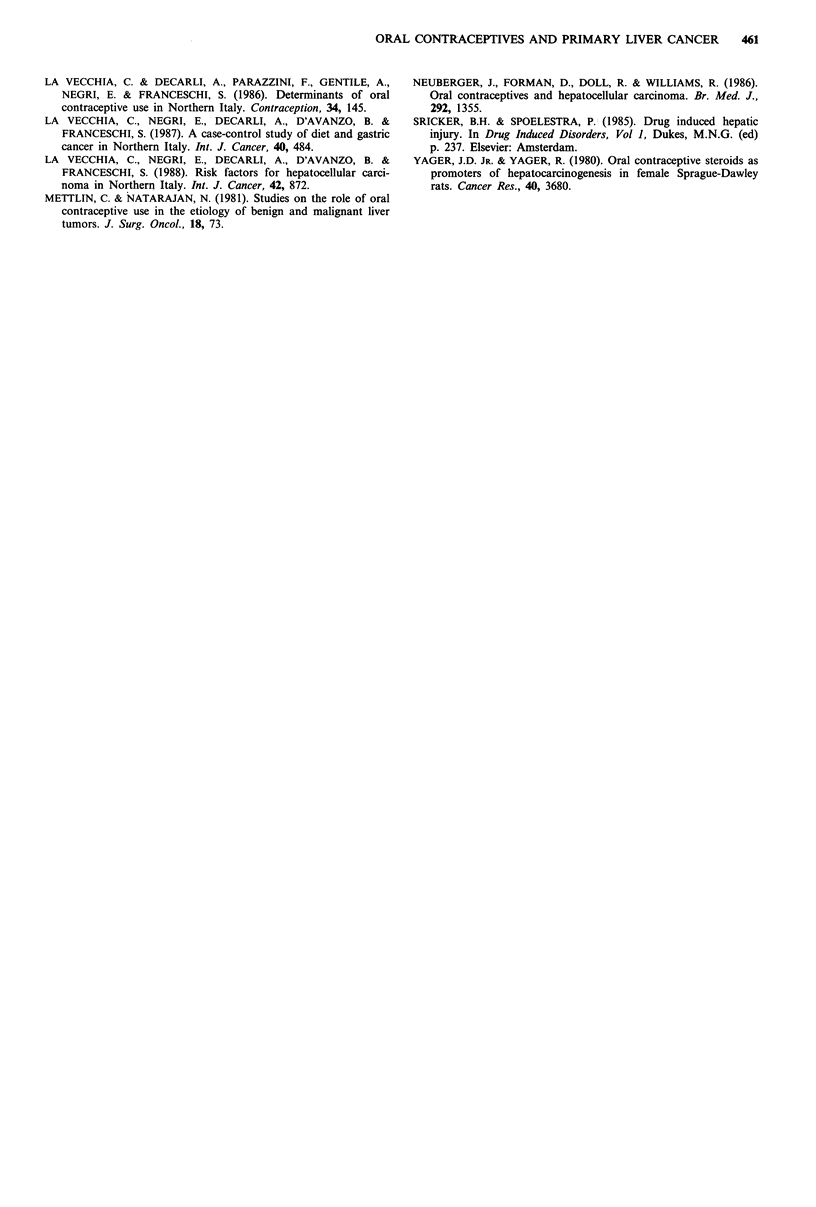


## References

[OCR_00133] Forman D., Doll R., Peto R. (1983). Trends in mortality from carcinoma of the liver and the use of oral contraceptives.. Br J Cancer.

[OCR_00142] Henderson B. E., Preston-Martin S., Edmondson H. A., Peters R. L., Pike M. C. (1983). Hepatocellular carcinoma and oral contraceptives.. Br J Cancer.

[OCR_00157] La Vecchia C., Decarli A., Parazzini F., Gentile A., Negri E., Franceschi S. (1986). Determinants of oral contraceptive use in northern Italy.. Contraception.

[OCR_00147] La Vecchia C., Decarli A. (1985). Trends in cancer mortality in Italy, 1955-1978.. Tumori.

[OCR_00162] La Vecchia C., Negri E., Decarli A., D'Avanzo B., Franceschi S. (1987). A case-control study of diet and gastric cancer in northern Italy.. Int J Cancer.

[OCR_00167] La Vecchia C., Negri E., Decarli A., D'Avanzo B., Franceschi S. (1988). Risk factors for hepatocellular carcinoma in northern Italy.. Int J Cancer.

[OCR_00172] Mettlin C., Natarajan N. (1981). Studies on the role of oral contraceptive use in the etiology of benign and malignant liver tumors.. J Surg Oncol.

[OCR_00138] Neuberger J., Forman D., Doll R., Williams R. (1986). Oral contraceptives and hepatocellular carcinoma.. Br Med J (Clin Res Ed).

[OCR_00177] Neuberger J., Forman D., Doll R., Williams R. (1986). Oral contraceptives and hepatocellular carcinoma.. Br Med J (Clin Res Ed).

[OCR_00187] Yager J. D., Yager R. (1980). Oral contraceptive steroids as promoters of hepatocarcinogenesis in female Sprague-Dawley rats.. Cancer Res.

